# Antifungal Treatment Duration in Hematology Patients With Invasive Mold Infections: A Real-life Update

**DOI:** 10.1093/ofid/ofae201

**Published:** 2024-04-09

**Authors:** Vera Portillo, Silvio Ragozzino, Elisavet Stavropoulou, Celine El-Khoury, Pierre-Yves Bochud, Frederic Lamoth, Nina Khanna, Dionysios Neofytos

**Affiliations:** Division of Infectious Diseases, University Hospital of Geneva, Geneva, Switzerland; Division of Infectious Diseases, University Hospital of Basel, Basel, Switzerland; Service of Infectious Diseases, Department of Medicine, Lausanne University Hospital and University of Lausanne, Lausanne, Switzerland; Service of Infectious Diseases, Department of Medicine, Lausanne University Hospital and University of Lausanne, Lausanne, Switzerland; Service of Infectious Diseases, Department of Medicine, Lausanne University Hospital and University of Lausanne, Lausanne, Switzerland; Service of Infectious Diseases, Department of Medicine, Lausanne University Hospital and University of Lausanne, Lausanne, Switzerland; Institute of Microbiology, Department of Laboratory Medicine and Pathology, Lausanne University Hospital and University of Lausanne, Lausanne, Switzerland; Division of Infectious Diseases, University Hospital of Basel, Basel, Switzerland; Division of Infectious Diseases, University Hospital of Geneva, Geneva, Switzerland

**Keywords:** acute myelogenous leukemia, antifungal treatment changes, antifungal treatment duration, invasive aspergillosis, invasive mold infections

## Abstract

**Background:**

Limited data exist on when and how to stop antifungal treatment (AFT) in patients with invasive mold infections (IMIs) who are immunocompromised.

**Methods:**

This retrospective multicenter study included adult patients with acute myelogenous leukemia and proven/probable IMI (1 January 2010–31 December 2022) in 3 university hospitals. The primary objective was to describe AFT duration and adaptation. Secondary objectives were to investigate the reasons for AFT adjustments and prolongation.

**Results:**

In total 71 patients with 73 IMIs were identified; 51 (71.8%) had an allogeneic hematopoietic cell transplant. Most infections were invasive aspergillosis (IA; 49/71, 69%), followed by mucormycosis (12, 16.9%) and other (12, 16.9%); there were 2 mixed infections. Median treatment duration was 227 days (IQR, 115.5–348.5). There was no difference in AFT duration between patients with IA and non-IA IMI (*P* = .85) or by center (*P* = .92). Treatment was longer in patients with an allogeneic hematopoietic cell transplant vs not (*P* = .004). Sixteen patients (22.5%) had no therapy modifications. In 55 patients (77.5%), a median 2 changes (IQR, 1–3; range, 1–8) were observed. There were 182 reasons leading to 165 changes, associated with clinical efficacy (82/182, 44.5%), toxicity (47, 25.8%), and logistical reasons (22, 12.1%); no reason was documented in 32 changes (18.8%). AFT was continued beyond days 90 and 180 in 59 (83%) and 39 (54.9%) patients, respectively, mostly due to persistence of immunosuppression.

**Conclusions:**

AFT in patients with acute myelogenous leukemia and IMI is longer than that recommended by guidelines and is frequently associated with treatment adjustments due to variable reasons. More data and better guidance are required to optimize AFT duration and secondary prophylaxis administration according to immunosuppression.

As easy as it may be to initiate empirical or targeted treatment for an invasive mold infection (IMI), discontinuing treatment may be a much more complex and difficult decision to make in clinical practice. Historically, antifungal treatment duration for IMI has been set at 12 weeks in the setting of clinical trials, with real-life data remaining scarce [1–9]. International guidelines and experts’ opinions suggest a minimum of 3 to 6 months of antifungal treatment in patients with invasive aspergillosis (IA) and mucormycosis, respectively, based on treatment response and the patient's net immunosuppression status, with European guidelines referring to treatment durations as long as 50 weeks [10–13]. However, treatment response and assessment of immune function remain poorly defined, allowing for wide interpretations and leading to variable treatment durations [1–3, 14]. Additionally, long treatment courses in complex patient populations, such as patients with hematologic malignancies and recipients of allogeneic hematopoietic cell transplant (HCT), may lead to frequent changes of antifungal agents due to reasons related to clinical efficacy and/or toxicities with potential impact on clinical outcomes [9]. In a single-center cohort of allogeneic HCT recipients with IMI, 2 changes of antifungal treatment on average (range, 0–8) were reported, with at least 1 antifungal change observed in 86% of cases [9]. Various reasons prompted those changes, such as clinical efficacy, toxicity, drug interactions, and logistical reasons. To further investigate the challenges associated with administration of antifungal treatment in a different high-risk patient population, we performed a retrospective study reviewing antifungal therapy courses of patients with acute myelogenous leukemia (AML) diagnosed with an IMI in 3 Swiss hematology centers.

## METHODS

### Study Design and Objectives

This observational retrospective multicenter cohort study comprised all consecutive adult patients (≥18 years old) with AML, diagnosed with a proven/probable IMI from 1 January 2010 to 31 December 2022. Patients were identified through the relevant institutional databases from 3 university hospitals in Switzerland (Basel, Geneva, Lausanne). Proven and probable IMIs were defined per consensus definitions [15]. For patients who had an allogeneic HCT, only infections diagnosed prior to the HCT were included. For patients with >1 IMI, only the first was considered. The study was approved by the local ethics committees. The primary objective was to describe the duration and number of changes of antifungal treatment. Treatment duration was defined as the time of uninterrupted mold-active agent administration starting with the diagnosis of an IMI. Given the retrospective nature of this study, it was not possible to differentiate between primary antifungal treatment and secondary antifungal prophylaxis, and both were included in the assessment of the overall duration of antifungal treatment for an IMI. For secondary objectives, we describe the reasons for antifungal treatment: adjustment (categorized as clinical efficacy, toxicity, and logistical reasons), continuation beyond 90 and 180 days, and discontinuation. Treatment selection reasons were arbitrarily divided in 3 major categories as previously described by our group and as assessed by the treating physicians and documented in the patients’ charts [1]. Clinical efficacy reasons prompting a specific treatment selection, as initial treatment or treatment change, included de-escalation for clinical improvement, escalation for clinical deterioration, targeted treatment for a clinical suspicion of IA or non-IA IMI, or a change attributed to subtherapeutic azole trough concentrations. Toxicity leading to treatment selection included liver or renal function impairment, neurotoxicity, or drug interactions. When a certain agent was selected as initial antifungal treatment based on an underlying pathology (eg, renal or liver impairment, potential drug interactions), toxicity was chosen as the reason of initial treatment selection rather than clinical efficacy. Logistical reasons consisted of changes due to insurance coverage or to facilitate patient discharge (eg, when changing intravenously to orally administered treatment).

### Data Collection

Pertinent data were retrospectively collected at all 3 centers through electronic medical records and entered into an electronic case report form stored on a REDCap electronic database [16, 17]. Data were collected for variables related to AML, IMI (antifungal prophylaxis administration within 30 days prior to IMI diagnosis, date of diagnosis, site of infection, pathogen), and antifungal treatment. Chest and/or sinus computed tomography and the following laboratory variables were collected at 90 and 180 days and at end of treatment (EOT): absolute neutrophil count, absolute lymphocyte count, CD4 count, platelet count, immunoglobulins, glomerular filtration rate, alanine aminotransferase, and γ-glutamyltransferase. Finally, chart documentation of treatment discontinuation at EOT by hematology and infectious disease services was recorded.

### Statistical Analysis

Study data were collected and managed by REDCap electronic data capture tools hosted at Geneva University Hospital. Descriptive statistics were used to characterize the study sample. Median and IQR were calculated for continuous variables and frequencies and percentages for categorical variables. Statistical analysis was performed with Stata (release 16; StataCorp), and figures were generated with Prism (version 8.0; GraphPad).

## RESULTS

### IMI Description

An overall 71 patients with AML were identified: 23, 20, and 28 at the university hospitals of Basel, Geneva, and Lausanne, respectively. In total there were 73 IMIs (mixed infection, n = 2) diagnosed at a median 31 days (IQR, 19−76) after AML diagnosis, without significant differences between IA and non-IA IMI (*P* = .59). From those patients, 51 (71.8%) underwent an allogeneic HCT at a median 98 days (IQR, 68–182) after IMI diagnosis. Antifungal prophylaxis during the last 30 days prior to IMI diagnosis was administered in 38 patients (53.5%) for a median 18 days (IQR, 13–24): fluconazole (n = 23, 60.5%), posaconazole (n = 13, 34.2%), and voriconazole (n = 2, 5.3%). Most infections were IA (49/73, 67%), followed by mucormycosis (n = 12, 16.5%) and other (n = 12, 16.5%); there were 2 mixed infections attributed to *Aspergillus fumigatus* and *Rhizomucor* spp and to *A fumigatus* and *Lichtheimia corymbifera.* Most IA cases were due to *A fumigatus* (23/49, 46.9%; including the 2 mixed infections), followed by *A flavus* (n = 2), *A terreus* (n = 2), *A terreus* and *A flavus* (n = 1), *A niger* (n = 1), and other unidentified *Aspergillus* spp (n = 3). The diagnosis of IA was based solely on a positive galactomannan enzyme immunoassay result in 17 of 49 (34.7%) cases. Twelve IMIs (16.9%) were caused by Mucorales (including the 2 mixed infections): *Rhizomucor* spp (n = 5), *Lichtheimia corymbifera* (n = 3), *Conidiobolus* (n = 2), and *Mucor* and a nonidentified Mucorales spp (n = 1 each). Another mold was identified in 12 of 71 (16.9%) patients: *Hormographiella aspergillata* (n = 3), *Penicillium* spp (n = 1), and nonidentified molds (n = 8). Most patients (59/71, 83.1%) had lung involvement, followed by sinus (n = 2, 2.8%), abdomen (n = 1, 1.4%), or multiple sites (n = 8, 11.3%). Of 73 IMIs, 25 (34.2%) were proven and 48 (65.7%) were probable. There were 16 of 24 (66.7%) proven non-IA IMIs and 9 of 49 (18.4%) proven IA IMIs (*P* < .001).

### Antifungal Treatment Duration

**Figure 1.**
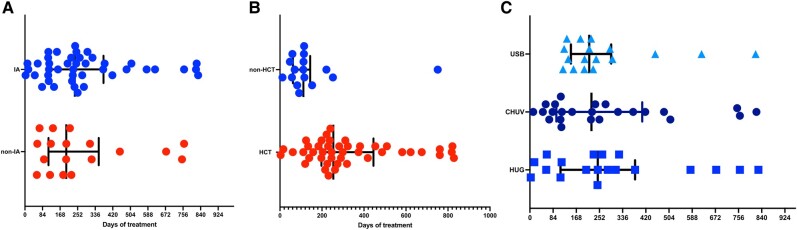
Presentation of antifungal treatment duration: *A*, type of invasive mold infection (IA vs not; *P* = .85); *B*, administration of an allogeneic HCT or not (*P* = .004); *C*, center (*P* = .92). Results are presented as whisker plots, with lines representing the median and 25% and 75% percentiles. CHUV, University Hospital of Lausanne; HCT, hematopoietic cell transplant; HUG, University Hospital of Geneva; IA, invasive aspergillosis; USB, University Hospital of Basel.

Antifungal treatment was started on day 0 (IQR, 0–2) of diagnosis, without difference in IA or non-IA IMI (*P* = .13; Table 1). Antifungal treatment with a single agent was initiated in almost all patients (n = 70, 98.6%), mainly with a mold-active azole (41/71, 57.7%), mostly voriconazole (38/41, 92.7%). Total treatment duration was a median 227 days (IQR, 115.5–348.5), which was discontinued because of treatment completion in 41 (67.2%) of 71 patients, death (n = 13, 18.3%), and palliative care (n = 2, 2.8%), while 5 (7%) patients were lost to follow-up. Median treatment duration for 56 patients alive at 12 weeks was 230 days (IQR, 122–348.5). There was no difference in median treatment duration between patients with IA (238.5 days; IQR, 115–374) and non-IA IMI (197.5 days; IQR, 120–319; *P* = .85; Figure 1*A*). Median treatment was significantly longer in patients who underwent an allogeneic HCT (254 days; IQR, 197.5–436) vs those without an HCT (111.5 days; IQR, 64.5–134.5; *P* = .004; Figure 1*B*). There was no significant difference in treatment duration by center (*P* = .92; Figure 1*C*).

**Table 1.** Antifungal Treatment Administered in 71 Patients With Acute Myelogenous Leukemia Diagnosed With Proven or Probable Invasive Mold Infections

	Median (IQR) or No. (%)			
	IMI (n = 71)	IA (n = 47)	Non-IA IMI (n = 24)^a^	*P* Value^b^
Treatment initiation, d	0 (0–2)	0.5 (0–2)	0 (0–3)	.16
Type of treatment^c^				.55
** **Monotherapy only	53 (74.6)	37 (78.7)	17 (70.8)	
** **Monotherapy and combination	17 (23.9)	10 (21.3)	7 (29.2)	
Type of initial treatment				.33
** **Monotherapy	70 (98.6)	47 (100)	23 (95.8)	
** **Combination therapy	1 (1.4)	0	1 (4.2)	
Initial agent administered^d^				.31
** **Mold-active azole	41 (57.7)	28 (59.6)	13 (54.2)	
** **Voriconazole	38	26	12	
** **Posaconazole	2	1	1	
** **Isavuconazole	1	1	0	
** **Liposomal amphotericin-B	27 (38)	16 (34)	11 (45.8)	
** **Echinocandin	3 (4.2)	3 (6.4)	1 (4.2)	
Reason for initial treatment selection^e^				
** **Clinical efficacy	56 (78.9)	34 (72.3)	23 (95.8)	.12
** **Toxicity	9 (12.7)	8 (17)	1 (4.2)	.25
** **Other^f^	6 (8.4)	6 (12.8)	0	>.99
Surgical intervention	26 (36.6)	12 (25.5)	14 (58.3)	.004
No. of treatment changes (range, 0–8)	2 (1–3)	2 (1–4)	2 (0–3)	.42
** **None	16	9	6	.76
** **1	10	5	5	
** **≥2	45	33	12	
Total treatment duration, d	227 (115.5–348.5)	238.5 (115–374)	197.5 (120–319)	.85
Treatment stop reason^g^				.15
** **Treatment completion	41 (67.2)	32 (72.7)	9 (53)	
** **Death	13 (21.3)	9 (20.5)	4 (23.5)	
** **Palliative care	2 (3.3)	1 (2.3)	1 (5.9)	
** **Loss to follow-up/other	5 (8.2)	2 (4.5)	3 (17.6)	

Data are presented as median (IQR) or No. (%).

Abbreviations: IA, invasive aspergillosis; IMI, invasive mold infection.

^a^There were 2 mixed infections due to *Aspergillus fumigatus* and Mucorales, which are considered in the non-IA group in this table.

^b^ *P* value compares IA vs non-IA.

^c^Information for 1 patient with non-IA IMI was limited to 3 days of monotherapy; additional information after day 3 was not available. This patient was included under monotherapy and accounted for a total treatment duration of 3 days.

^d^One patient with non-IA IMI was treated with combination therapy including liposomal amphotericin-B and an echinocandin.

^e^Clinical efficacy reasons prompting a specific treatment selection, either as initial treatment or as treatment change, included de-escalation for clinical improvement, escalation for clinical deterioration, targeted treatment for a clinical suspicion of IA or non-IA IMI, or a change due to subtherapeutic azole trough concentrations. Toxicity leading to treatment selection included liver or renal function impairment, neurotoxicity, or drug interactions. When a certain agent was selected as an initial antifungal treatment based on an underlying pathology (eg, renal or liver impairment, potential drug interactions), toxicity, rather than clinical efficacy, was chosen as the reason of initial treatment selection. Other reasons included changes due to insurance coverage or to facilitate patient discharge (eg, when changing from intravenously to orally administered treatment).

^f^Concomitant invasive candidiasis (n = 1), neutropenic enterocolitis (n = 1), and unknown reason (n = 4).

^g^Information was available for 61 patients overall.

### Antifungal Treatment Changes

**Figure 2.**
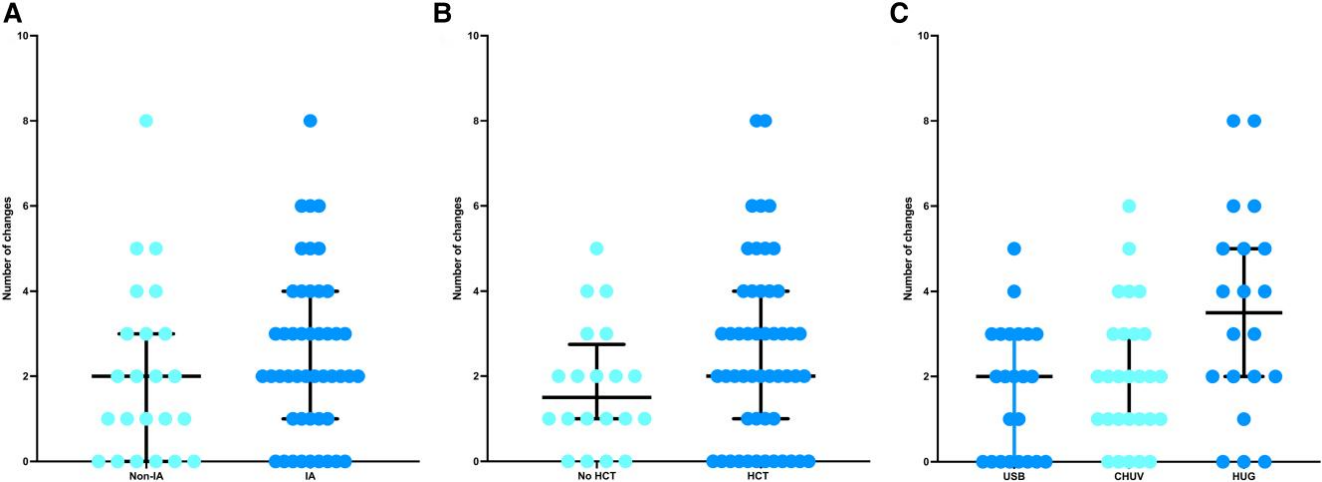
Presentation of antifungal treatment number of changes: *A*, diagnosis of invasive mold infection (IA vs not; *P* = .42); *B*, administration of an allogeneic HCT or not (*P* = .12); *C*, center (*P* = .001). Results are presented as whisker plots, with lines representing the median and 25% and 75% percentiles. CHUV, University Hospital of Lausanne; HCT, hematopoietic cell transplant; HUG, University Hospital of Geneva; IA, invasive aspergillosis; USB, University Hospital of Basel.

Overall, 16 (22.5%) of 71 patients did not encounter any changes in their treatment from the beginning until EOT (Table 1). In the remaining 55 patients, antifungal treatment was changed on average twice (IQR, 1–3), with a range from 1 to 8. There were no significant differences in the number of changes between patients with IA and non-IA IMI (*P* = .42) or an allogeneic HCT and not (*P* = .12; Figure 2*A* and 2*B*). There were more changes observed in Geneva (median, 3.5; IQR, 2–5) vs Basel (2; IQR, 0–3) and Lausanne (2; IQR, 1–3; *P* = .004; Figure 2*C*).

### Antifungal Adjustment Reasons

There were 182 reasons leading to 165 antifungal treatment adjustments (Table 2). Treatment changes were prompted by clinical efficacy (82/182, 44.5%), toxicity (n = 47, 25.8%), and logistical reasons (n = 22, 12.1%); a reason was not documented in 32 (18.8%) changes. Clinical efficacy reasons consisted of targeted treatment administration (21/82, 25.9%), treatment escalation owing to clinical/radiologic progression (n = 20, 24.7%), clinical suspicion for non-IA IMI (n = 14, 17.3%) or IA (n = 6, 7.4%), treatment de-escalation for clinical improvement (n = 7, 8.6%), and subtherapeutic therapeutic drug monitoring (n = 5, 6.2%). No significant differences were noted in the number of changes attributed to clinical efficacy across the 3 major antifungal classes (*P* = .97). Toxicity leading to treatment changes included potential drug interactions (17/47, 36.2%), hepatotoxicity (n = 16, 34%), and nephrotoxicity (n = 6, 12.8%). Azoles (28/47, 59.6%) were more likely to be associated with toxicities, predominately hepatotoxicity and drug interactions (*P* = .006). Most drug interactions were potential interactions with conditioning regimens (n = 10), leading to treatment changes from an azole to an echinocandin. Among 22 changes prompted for logistical reasons, 18 (81.8%) were due to changing from an intravenously to orally administered agent.

**Table 2.** Reasons Leading to Changes of Antifungal Treatment in 71 Patients With Acute Myelogenous Leukemia With Proven/Probable Invasive Mold Infections

	Reason, No. (%)								
	Overall (n = 182)	Azoles (n = 80)	Echinocandins (n = 21)	L-AMB (n = 56)	Combination (n = 25)	*P* Value^a^	VCZ (n = 51)	PCZ (n = 19)	IVC (n = 10)
Clinical efficacy	81 (44.5)	35 (43.7)	8 (38.1)	24 (42.8)	14 (56)	.97	20 (39.2)	9 (47.4)	6 (60)
Targeted treatment	21 (25.9)	4 (11.4)	4 (50)	7 (29.2)	6 (42.9)		1	3	0
Clinical suspicion for IA	6 (7.4)	0	3 (37.5)	3 (12.5)	0		0	0	0
Clinical suspicion for non-IA IMI	14 (17.3)	9 (25.7)	1 (12.5)	3 (12.5)	1 (7.1)		5	3	1
Clinical/radiologic progression	20 (24.7)	13 (37.2)	0	6 (25)	1 (7.1)		7	2	4
Clinical/radiologic improvement	7 (8.6)	1 (2.9)	0	4 (16.6)	2 (14.4)		1	0	0
Subtherapeutic TDM	5 (6.2)	4 (11.4)	0	0	1 (7.1)		3	1	0
Other^b^	8 (9.9)	4 (11.4)	0	1 (4.2)	3 (21.4)		3	0	1
Toxicity	47 (25.8)	28 (35)	9 (42.9)	8 (14.3)	2 (8)	.006	21 (41.2)	5 (26.3)	2 (20)
Drug interactions^c^	17 (36.2)	14 (50)	3 (33.3)	0	0		10	3	1
Hepatotoxicity	16 (34.0)	10 (35.7)	3 (33.3)	2 (25)	1 (50)		9	1	0
Nephrotoxicity	6 (12.8)	1 (3.6)	0	4 (50)	1 (50)		1	0	0
Other^d^	8 (17.0)	3 (10.7)	3 (33.3)	2 (25)	0		1	1	1
Logistical reasons	22 (12.1)	3 (3.8)	2 (9.5)	16 (28.6)	1 (4)	<.001	0	1 (5.3)	2 (20)
IV to PO	18 (81.8)	0	1 (50)	16 (100)	1 (100)		0	0	0
Insurance coverage	1 (4.6)	1 (33.3)	0	0	0		0	0	1
Other^e^	3 (13.6)	2 (66.7)	1 (50)	0	0		0	1	1
Unknown/not reported	32 (17.6)	14 (17.5)	2 (9.5)	8 (14.3)	8 (32)	.70	10 (19.6)	4 (21)	0

Abbreviations: IA, invasive aspergillosis; IMI, invasive mold infection; IV, intravenous; IVC, isavuconazole; L-AMB, liposomal amphotericin-B; PCZ, posaconazole; PO, oral; TDM, therapeutic drug monitoring; VCZ, voriconazole.

^a^ *P* value compares azoles, echinocandins, and liposomal amphotericin-B.

^b^Other clinical efficacy reasons included suspicion of coinfection (n = 3), antifungal resistance suspected/confirmed (n = 2), target trough azole level achieved (n = 1), change to secondary prophylaxis (n = 1), and low clinical suspicion of aspergillosis (n = 1).

^c^Drug interactions included conditioning-associated interactions (n = 10), chemotherapy-associated interactions (n = 1), aprepitant coadministration (n = 1), and not specified (n = 5).

^d^Other toxicity reasons included neurotoxicity (n = 1), gastrointestinal tract intolerance (n = 1), allergic reaction (n = 1), supratherapeutic TDM (n = 1), chills (n = 1), and unknown (n = 3).

^e^Other logistical reasons included isavuconazole out of stock (n = 1), patient discharge (n = 1), and unknown (n = 1).

### Antifungal Treatment Prolongation Until EOT

Clinical and immunologic variables at EOT are presented in Table 3. Among 60 patients with available data at EOT, treatment was discontinued in 41 (68.3%) for treatment completion vs 15 (25%) due to death/palliative care. Median white blood cell, absolute lymphocyte, and CD4 counts at EOT were 4.6 × 10^3^/mm^3^ (IQR, 3–3.6), 0.9 × 10^3^/mm^3^ (IQR, 0.5–1.5), and 232.5 cells/µL (IQR, 113–385), respectively. A minority of patients were still undergoing immunosuppressive treatment, such as corticosteroids at a prednisone dose >10 mg daily (13/60, 21.6%). Antifungal treatment was continued beyond day 90 in 59 (83%) patients. A lack of clinical or radiologic response was documented in a small number of those patients (n = 16, 27.1%). In contrast, treatment was prolonged mostly because of persistence of immunosuppression, whether due to continuation of immunosuppressive treatment administration (n = 33, 55.9%), an allogeneic HCT (n = 28, 47.4%), or AML relapse (n = 2, 3.4%). Similarly, more than half of patients (n = 39, 54.9%) continued their treatment after 180 days owing to continuation of immunosuppressive treatment administration (n = 24, 61.5%), allogeneic HCT (n = 11, 28.2%), or AML relapse (n = 2, 5.1%). All patients had a documented clinical or radiologic response at 180 days. Chart documentation of treatment discontinuation was observed in 39 (65%) patients, although a discussion between hematology and infectious disease services or a dedicated infectious disease consultation at the time of treatment discontinuation was noted in 25 (41.6%) and 20 (33.3%) cases, respectively.

**Table 3.** Clinical and Immunologic Parameters at EOT and for Patients Whose Treatment Continued Beyond Day 90 and 180

	Median (IQR) or No. (%)^a^		
	EOT (n = 60)	90 d (n = 59)	180 d (n = 39)
Treatment duration, d			
** **IA	238.5 (115–374)	245.5 (155.5–377.5)	278 (237–418)
** **Non- IA	197.5 (120–319)	215 (134–454)	315 (198–675)
Treatment continuation reasons^b^			
** **Lack of clinical/radiographic response	…	16 (27.1)	0
** **Continued immunosuppressive treatment	…	33 (55.9)	24 (61.5)
** **Allogeneic hematopoietic cell transplant	…	28 (47.4)	11 (28.2)
** **Disease relapse	…	2 (3.4)	2 (5.1)
** **Unknown	…	6 (10.2)	5 (12.8)
Treatment discontinuation reasons			
** **Treatment completion	41 (68.3)	…	…
** **Death/Palliative care	15 (25)	…	…
** **Unknown	4 (6.7)	…	…
Laboratory tests^c^			
** **White blood cell count, × 10^3^/mm^3^	4.6 (3.3–6)	3.9 (2–5.8)	4 (3–5.5)
** **Absolute neutrophil count, × 10^3^/mm^3^	2.9 (1.6–4.3)	2.5 (1–3.9)	2.5 (1.6–3.4)
** **Absolute lymphocyte count, × 10^3^/mm^3^	0.9 (0.5–1.5)	0.6 (0.4–0.9)	0.7 (0.3–1.1)
** **Platelet count, × 10^3^/mm^3^	91.5 (18.5–161.5)	113 (45–149)	118 (36–165)
** **CD4 count, cells/µL	232.5 (113–385)	23.5 (10–100)	80 (44–97)
** **Immunoglobulin G, g/L	6 (5.2–9.3)	6.6 (4.8–8.9)	6 (5.5–8.5)
** **Aspartate aminotransferase, IU/L	27.5 (19–44)	24.5 (14–39)	17 (14–30)
** **γ-Glutamyltransferase, IU/L	113 (48–215)	102 (66–252)	67 (50–163)
** **Glomerular filtration rate, mL/min/m^2^	60 (60–90)	60 (60–94)	71 (60–95)
Immunosuppression			
** **Corticosteroids^d^	13 (21.6)	7 (13.3)	11 (36.7)
** **Other immunosuppressive treatment	22 (36.7)	27 (45.8)	22 (73.3)
Computed tomography			
** **Chest	40 (66.6)	…	…
** **Sinus	4 (6.7)	…	…
Chart documentation			
** **Treatment discontinuation	39 (65)	…	…
** **Discussion between hematology and ID	25 (41.6)	…	…
** **ID consultation	20 (33.3)	…	…

Abbreviations: EOT, end of treatment; IA, invasive aspergillosis; ID, infectious diseases.

^a^Data were not available for all patients at EOT or by day 90 and 180 after treatment initiation.

^b^Treatment continuation reasons were not mutually exclusive, meaning that treatment could have been continued due to >1 reason per patient. Allogeneic hematopoietic cell transplant and disease relapse were included as part of continued immunosuppressive treatment.

^c^The following were the only statistically significant results comparing laboratory variables: EOT vs day 90 for absolute lymphocyte count, *P* = .008; EOT vs day 180 for absolute lymphocyte count, *P* = .05; EOT vs day 90 for CD4 count, *P* = .04; EOT vs day 180 for CD4 count, *P* = .02. All other comparisons were not statistically significant and are not presented on this table.

^d^Corticosteroids included administration of daily dose of prednisone >10 mg.

### Mortality

**Figure 3.**
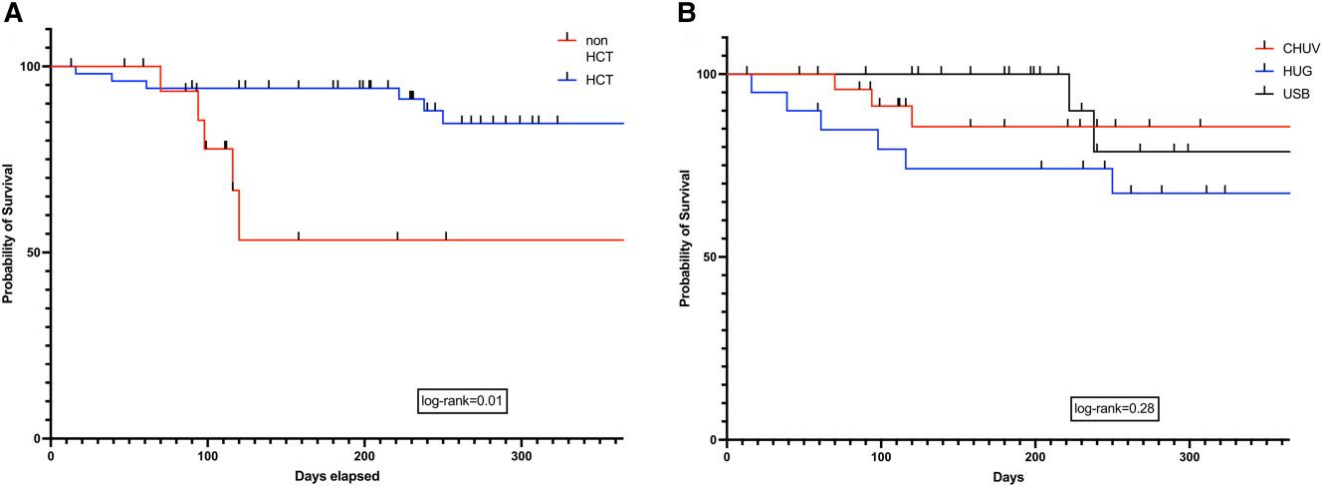
Kaplan-Meier survival curves depicting all-cause mortality at 1-year after IMI diagnosis in patients: *A*, administration of an allogeneic HCT or not (log-rank test, *P* = .01); *B*, center (log-rank test, *P* = .28). CHUV, University Hospital of Lausanne; HCT, hematopoietic cell transplant; HUG, University Hospital of Geneva; USB, University Hospital of Basel.

There was no difference in all-cause 12-week mortality in patients with IA and non-IA (log-rank test, *P* = .78), proven and probable IMI (log-rank test, *P* = .78), or surgery or not (log-rank test, *P* = .20). All-cause 1-year mortality after IMI diagnosis was 18.3% (13/71 patients): 15.7% (8/51) and 25% (5/20) in patients with and without an allogeneic HCT, respectively (log-rank test, *P* = .01; Figure 3*A*). There was no difference in all-cause 1-year mortality across the 3 centers participating in the study (log-rank test, *P* = .28; Figure 3*B*).

## DISCUSSION

This retrospective multicenter study demonstrates the complexity and challenges associated with the treatment of IMI in high-risk populations, such as patients with AML. Our observations suggest that prolonged treatment courses are frequently administered in hematology patients with IMI. We report that treatment duration was on average >6 months, at times as long as 1 year, regardless of the type of IMI or treating center. This is consistent with previous real-life data reported in allogeneic HCT recipients [9]. In fact, treatment was much longer in patients who underwent an allogeneic HCT vs patients with AML who did not undergo transplantation. In the latter, treatment might have been discontinued because they died before an HCT or they had a favorable AML prognosis with treatment completion by the end of chemotherapy with resolution of neutropenia, the major risk factor in this patient group. In contrast, patients with AML and a subsequent allogeneic HCT might have continued treatment due to prolonged severe immunosuppression associated with their underlying malignancy, conditioning regimen, and posttransplant complications such as graft-vs-host disease (GvHD) and treatment. As is well known, HCT recipients with GvHD undergoing treatment with high-dose corticosteroids or other immunosuppressive therapies are at higher risk for IMI, including primary or relapsing infections. This might be one of the reasons for continuing antifungal treatment in this subgroup of patients. In fact, treatment was continued beyond 90 and 180 days in most patients owing to transition to an allogeneic HCT and/or continuation of an administered immunosuppressive treatment. Considering the large number of patients with a clinical response in our series, treatment prolongation was predominately based on persistent immunosuppression. This is consistent with current guidelines suggesting that treatment discontinuation be tailored by clinical response and the patient's immunosuppression status, although this combination has never been validated in the context of clinical trials, where a duration of 12 weeks of treatment has been historically and uniquely studied [4–8, 10–13, 18].

Prolonging administration of antifungal agents after treatment completion may represent secondary prophylaxis, used to prevent infection relapse in states of persistent immunosuppression [18]. Although a term frequently used, secondary antifungal prophylaxis remains poorly defined and requires clarification in terms of administration (when, how, and for how long) [18]. Notably, international guidelines suggest that secondary prophylaxis be administered in patients with continued immunosuppression. However, we are lacking clear-cut definitions for what are the degree and duration of immunosuppression that would warrant prolongation of antifungal agent administration. This is a field that requires continuous efforts and research to optimally define the net state of immunosuppression that would be considered safe enough to discontinue antifungal treatment or prevention. Given the retrospective nature of this study, it was not feasible to discern from patients’ charts at which point treatment was transitioned to secondary prophylaxis. In a recent cross-sectional internet-based questionnaire survey from Europe, the majority of clinicians treating hematology patients with IMI employed secondary prophylaxis, in most cases until the end of the immunosuppressive regimen [3]. The study clearly showed that, whether it refers to antifungal treatment or secondary prophylaxis, treatment interruption remains problematic for most physicians, who would appreciate an algorithm to help decision making in the setting [3].

Notably and although suggested by guidelines and commonly used in clinical practice, immune status assessment remains loosely defined. In addition to the administration of immunosuppressive treatments such as chemotherapy and GvHD prophylaxis or treatment, laboratory tests may occasionally be used to help clinicians better evaluate the net immune status of their patients. For instance, CD4 counts have been routinely used in the evaluation of patients with HIV as a surrogate of their immune function. We reviewed white blood cell, absolute neutrophil, absolute lymphocyte, platelet, and CD4 counts as well as immunoglobulins of patients treated for an IMI at EOT and by 3 and 6 months postdiagnosis. Although most patients had robust white blood cell, absolute neutrophil, and platelet counts, they remained lymphopenic even at EOT. However, higher absolute lymphocyte and CD4 counts were noted at EOT as compared with day 90 and 180 after treatment initiation. The latter may merely represent the natural evolution of lymphocyte count reconstitution at a distance posttransplant. There are no current data on the threshold of absolute lymphocyte or CD4 count or the doses of immunosuppressive treatment beyond which treatment could be safely discontinued. This is a field where more data are needed to better define easy-to-use tools of immune reconstitution evaluation in the decision-making process of antifungal treatment discontinuation.

Most patients in our cohort had at least 1 treatment change, with a range of up to 8 changes during their treatment, similar to findings reported by Roth et al [9]. Variability was observed in the number of changes reported at different centers, likely reflecting differences in patient populations and local clinical practices. No significant differences were observed in the number of changes between IA and non-IA IMI, although there was a trend for fewer changes in AML cases without an allogeneic HCT as compared with those with one. This could be, in part, attributed to the longer survival of the latter patient group and the potential drug interactions between azoles and conditioning regimens observed in transplant recipients. In fact, drug interactions accounted for most toxicity-related causes that prompted treatment changes.

This study has many limitations, such as the small number of patients, retrospective design, and lack of data for all patients at all time points. In addition, information on neutropenia duration was not collected or reported. However, considering the long duration of antifungal treatment administration, it is less likely that the latter was associated to persistence of neutropenia but rather the other factors listed in this study, including administration of immunosuppressive treatment due to an allogeneic HCT and/or GvHD. Yet, it remains one of the few real-life studies describing the complexities and current issues in antifungal treatment of IMI in high-risk patients. Our data point to the urgent need for better tools, definitions, and clinical algorithms to support clinicians to decide when and how to stop antifungal treatment. Of particular interest remains the field of assessing the clinical response and immune status of high-risk patients treated for IMI and the definition of treatment vs secondary prophylaxis in that context. Finally, new antifungal treatment options are needed to improve safety, tolerability, and clinical outcomes.

## Notes

***Acknowledgments.*** We thank all patients and the nursing and other staff involved in the care of our patients.

***Author contributions*.** D. N. conceived the idea of the study and the principal study design and contributed to manuscript writing and revision. V. P. was responsible for data collection and analysis and contributed to table and figure creation and manuscript writing and revision. All other authors contributed to data collection and manuscript revision.

***Data availability.*** The data sets analyzed for this study are available from the corresponding author upon request.

***Disclaimer.*** None of the funding sources were involved in study de­sign and conduct; patient recruitment; data collection, analysis, and inter­pretation; writing of the manuscript; or decision to submit the article for publication.

***Patient consent.*** All patients provided written consent for data utilization, and the study was approved by local ethical committees (Geneva Ethics Committee 2020-01072).

***Financial support*.** This work was supported by a research grant to the Fungal Infection Network of Switzerland, which receives unrestricted funds from Pfizer, Gilead, and Merck, Sharp & Dohme.
